# Impact of neutropenia on delivering planned adjuvant chemotherapy: UK audit of primary breast cancer patients

**DOI:** 10.1038/sj.bjc.6601279

**Published:** 2003-11-25

**Authors:** R C F Leonard, D Miles, R Thomas, F Nussey

**Affiliations:** 1 Cancer Institute Singleton Hospital, Swansea SA2 8QA, UK; 2 Guy's Hospital, London SE1 9RT, UK; 3 St Thomas' Hospital, London SE1 7EH, UK; 4 Bedford Hospital NHS Trust, Bedford MK42 9DJ, UK; 5 Addenbrooke's NHS Trust, Cambridge CB 2QQ, UK; 6 Western General Hospital, Edinburgh EH4 2XU, UK

**Keywords:** adjuvant chemotherapy, breast cancer, dose intensity, neutropenia

## Abstract

The UK audit was undertaken in primary breast cancer patients receiving adjuvant chemotherapy to: (1) record the incidence of neutropenic events (hospitalisation due to febrile neutropenia, dose delay of ⩾1 week or dose reduction of ⩾15% due to neutropenia); (2) evaluate the impact of neutropenic events on overall dose intensity (DI) received and (3) review the use of granulocyte colony-stimulating factor (G-CSF) in clinical practice. Data from 422 patients with Stage I–III breast cancer were collected from 15 centres. Cyclophosphamide, methotrexate and 5-fluorouracil(CMF)- or anthracycline-based regimens were the most commonly used. Only 5.2% of patients received G-CSF. Overall, 29% of patients experienced a neutropenic event, most frequently dose delay. Neutropenic events had a significant impact on the ability to deliver planned DI. Out of 422 patients, 17% did not achieve 85% of their planned DI; due to neutropenia in 11% of patients. Of the neutropenic patients receiving CMF- or anthracycline-based regimens, around 40 and 32% of patients, respectively, did not achieve 85% of their planned DI. Patients who experienced one neutropenic event had a higher risk of a second event. During adjuvant chemotherapy of primary breast cancer, neutropenic events are common, likely to occur in subsequent chemotherapy cycles, and have a significant impact on receiving planned DI.

Adjuvant chemotherapy is a well-established treatment modality for patients with high-risk primary breast cancer, and has a beneficial impact on survival (Early Breast Cancer Trialists' Collaborative Group, 1998). In order to obtain this benefit, maintaining scheduled chemotherapy dose intensity (DI) is an important factor: both retrospective and prospective studies suggest that as DI is decreased, treatment outcome may be compromised. Bonadonna *et al* (1995) reviewed the results of therapy based on the percentage of projected dose of cyclophosphamide, methotrexate and 5-fluorouracil (CMF) received in the first Milan adjuvant trial, which involved over 300 patients. Survival was worse in those patients who received less than 85% of the projected dose of treatment (Bonadonna *et al*, 1995). Furthermore, patients who received <65% of their projected dose had similar survival rates as patients who received no chemotherapy at all. This was, however, a retrospective analysis and some of these patients may have performed poorly because of confounding factors such as medically significant comorbidities, which also may have had an impact on survival.

Prospective studies have evaluated the impact of chemotherapy dose and DI on clinical outcome. The CALGB 8541 study evaluated three different DI levels (low, moderate and high) of cyclophosphamide, adriamycin and fluorouracil (CAF) in over 1500 women with node-positive Stage II breast cancer (Wood *et al*, 1994). One group received cyclophosphamide 400 mg m^−2^ of body-surface area and 40 mg m^−2^ of adriamycin once every 28 days and 400 mg m^−2^ of fluorouracil twice every 28 days, for six cycles. Another group received 50 percent higher doses of the three drugs (600, 60 and 600 mg, respectively) but for only four cycles, so that the total dose was identical in these two groups, but the DI was higher in the second. The third group received half the total dose used in the other two groups and at half the DI used in the second group. Overall survival (OS) at 9-year follow-up was significantly improved in those women receiving CAF at moderate or high DI *vs* those receiving a lower DI (Budman *et al*, 1998). The lowest dose level in the trial was therefore likely to have been below the minimum effective threshold dose and above this level there was little evidence of a DI effect. Data from a 5-year follow-up study of women with poor prognosis, node-positive breast cancer also suggested that OS and disease-free survival were significantly improved in women receiving FEC regimens (fluorouracil, epirubicin and cyclophosphamide) containing a higher dose of epirubicin (100 mg m^−2^) compared with those receiving regimens containing only 50 mg m^−2^ of epirubicin (The French Adjuvant Study Group, 2001).

Despite the evidence that indicates the benefits of maintaining planned DI, doses are often reduced or delayed in clinical practice. This is undertaken to minimise the toxic sequelae of treatment in patients whose blood counts have not adequately recovered (Hayes, 2001). Although such dose reductions often result in minimal decreases in toxicity, they may have a significant impact on reducing the relative DI (RDI) of treatment received (Hayes, 2001). As a result, survival outcomes may be compromised. For example, if a patient is due to receive six cycles of chemotherapy and on cycle two, doses are reduced by 25%, assuming that this level of dose reduction is maintained, the patient will only have received 79% of the initial planned dose at the completion of therapy (Table 1a

**Table 1a:** Impact of dose reductions on dose delivered

	**Dose reduction (%)**			
	**No dose reduction**	**20**	**25**	**50**
	**Dose delivered^a^(% planned)**			
Cycle 1	100	**80**	**75**	**50**
Cycle 2	100	**83**	**79**	**58**
Cycle 3	100	87	**83**	**67**
Cycle 4	100	90	88	**75**
Cycle 5	100	93	92	**83**
Cycle 6	100	97	96	92

a Assuming that this level of dose reduction is maintained until completion of therapy.

Bold values signify that survial outcomes may be compromised.

). Similarly, a delay in chemotherapy delivery reduces the DI received (Table 1b

**Table 1b:** Impact of dose delay on dose intensity

**Days delay over course**	**% projected dose intensity^a^**
0	100
7	95
14	90
21	86
28	82
35	78
42	75

a Assumes that full doses were given in each cycle. Thus, a 1-week delay lowers the % projected dose intensity by approximately 5%.

). It has been calculated that each 7-day delay results in an approximate 5% decrease in the DI (Longo *et al*, 1991).

In addition to dose modifications, severe neutropenia can lead to febrile episodes, and to the possible requirement for hospitalisation and i.v. antibiotic treatment. Neutropenia, therefore, also has an impact upon both morbidity and the cost of treatment (Seifeldin and Schultz, 1998). There are variations in the degree of neutropenia experienced, depending on the chemotherapy regimen used (Gurney, 1989). However, there are limited data to indicate the number of breast cancer patients actually affected by severe neutropenia in clinical practice, and the magnitude of its effect on overall DI received (Dranitsaris and Tran, 1995; Hayes, 2001). For example, we are not aware of any previous data relating to patients treated within the UK. The UK neutropenia audit was initiated to evaluate the extent to which neutropenic complications were encountered in clinical practice and affect the ability to deliver planned chemotherapy in the adjuvant treatment of primary breast cancer. As there is evidence that the chemotherapy regimen employed is an important factor relative to outcome (Levine *et al*, 1998), the range of regimens currently employed was recorded in this audit.

The use of granulocyte colony-stimulating factor (G-CSF), which stimulates neutrophil recovery after chemotherapy and thus alleviates drug-induced neutropenia, may allow the delivery of chemotherapy as scheduled in patients with breast cancer (de Graaf *et al*, 1996). Data regarding the extent and impact of the use of G-CSF in the adjuvant treatment of primary breast cancer in the UK are also limited. This was also evaluated in this audit.

## MATERIALS AND METHODS

### Patients and centres

Both district general and teaching hospitals across the UK, with a breast cancer practice and available staff to complete records, were asked to participate in the audit. Data were collected in relation to patients in whom adjuvant chemotherapy for primary breast cancer (Stages I–III) was being initiated. We aimed to involve 15 centres and collect data from 400 patients.

### Data collection

Generally, data were collected prospectively using either a preprogrammed psion palm top computer or paper records. Patient details were recorded, including height, weight, date of birth, diagnosis, stage of disease and prior or concomitant radiotherapy (yes/no). The planned treatment protocol was recorded prior to the start of therapy and the actual treatment given were then recorded for each cycle, after it had been administered. Any delay in administration of chemotherapy and the reasons for this were recorded, as was the absolute neutrophil count (ANC) at the time of the delay, and whether the patient had experienced neutropenia during that cycle. A ‘trigger’ ANC at which doses were delayed or reduced was not specified in the protocol. As the audit reflects current clinical practice, ANC thresholds for dose delay/reduction varied from centre to centre. When the dose of chemotherapy administered was less than that planned, the reason for the dose reduction and the percentage of planned dose given were recorded.

All hospitalisations, their causal relation to neutropenia, the temperature and ANC on admission, and the length of hospital stay were recorded. Use of haematopoietic growth factors (dose, frequency of administration, total number of doses and reason for administration) was also recorded.

In some centres, prospective data collection was not possible and data were collected retrospectively from patient notes (40%).

Data were collected for the cycles up until a patient discontinued or switched therapy and the RDI calculated relative to what they would have received up until that time. Data collected from each centre were entered onto a central database. Each patient record was checked for anomalies in data entry or any missing data and these were referred back to the centre for validation.

### Data evaluation

The primary objective of the audit was to record the incidence of neutropenic events and to evaluate their impact on RDI received. Secondary objectives were to evaluate the impact of a neutropenic event on the risk of further neutropenic events in subsequent cycles, and to record the use of G-CSF in clinical practice.

Neutropenic events were defined as: hospitalisation due to febrile neutropenia; dose delay of ⩾1 week due to neutropenia; and/or dose reduction of ⩾15% due to neutropenia.

Dose intensity is defined as the amount of drug delivered per unit time, generally expressed as mg m^−2^ week^−1^, and RDI is the amount of drug (or average amount of drugs for a combination regimen) delivered per unit time compared with an arbitrarily chosen regimen (Hryniuk, 1998). In this audit, RDI was determined as the decimal fraction of the ratio of the regimen delivered to the planned regimen, as follows:


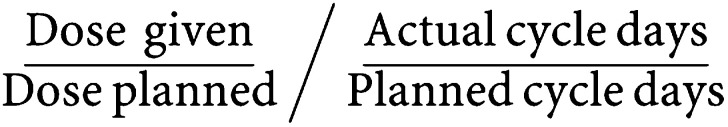


### Statistical analyses

Data are presented as descriptive statistics. Statistical analysis of the impact of neutropenic events on RDI and a comparison of received RDI of patients who did and did not experience neutropenic events were performed using either a one-tailed *z*-test or a two-tailed *t*-test. Values in the one-tailed *z*-test were considered significant at the 5% level (*P*<0.05) if the test statistic was greater than 1.64 standard errors (s.e.) above the mean. Values in the two-tailed *t*-test were considered significant at the 5% level (*P*<0.05) if the test statistic was greater than 1.96 s.e. either above or below the mean.

## RESULTS

### Patients and centres

The period of data collection was from June 1997 to June 1999. Data were collected in relation to 422 patients with Stage I – III breast cancer from 15 centres, including both district general and teaching hospitals across the UK.

Characteristics of the patients included in the audit are presented in Table 2

**Table 2:** Baseline demographic characteristics of patients in the audit

**Characteristic**	**Mean (range)**
Age (years)	49.3 (22.8–68.8) (*n*=408)^a^
Height (cm)	161.8 (131–198) (*n*=380)^a^
Weight (kg)	69.9 (40–131) (*n*=390)^a^
	
*Stage of disease*	
I	80
II	254
III	88
	
*Prior radiotherapy*	
Yes	8%
No	91% (1% unknown)^a^
	
*Concomitant radiotherapy*	
Yes	36%
No	64%
	
Number of positive nodes	3.17 (0–33) (*n*=398)^a^
	(median value=1.00)

a Data not available for all 422 patients enrolled in the audit.

. The majority of patients had Stage II disease and the median number of positive nodes was 1.

A wide variety of chemotherapy regimens was administered (Table 3a

**Table 3a:** Breast cancer patients Stages I – III by regimen

	**Patients**	**Stage I**	**Stage II**	**Stage III**	**Total**
**Regimens**	**%**	***n***	***n***	***n***	***n***
CMF (oral) day 1 – 8	28	29	76	14	119
CMF (i.v.) day 1 – 8	16	10	35	22	67
CMF (3 weekly)	17	15	50	6	71
AC	12	4	31	14	49
Adriamycin/CMF	11	1	26	18	45
FEC	8	19	16	1	36
NEAT	3	2	8	3	13
Adriamycin	2	0	7	2	9
CAF	1	0	3	4	7
Epirubicin/CMF	<1	0	2	1	3
Other	<1	0	0	3	3
					
Total	100	80	254	88	422

CMF=Cyclophosphamide, methotrexate and 5-fluorouracil; AC=anthracydine; FEC=fluorouracil, epirubicin and cyclophosphamide; CAF=cyclophosphamide, adriamycin and fluorouracil. Other=epirubicin, epirubicin/cyclophosphamide or docetaxel.

and 3b

**Table 3b:** Details of planned chemotherapy regimens used in the audit

	**Drug (dose (mg m^−2^)/administration route/days given/(cycle length))**				
**Regimen**	**Cyclophosphamide**	**Methotrexate**	**5-FU**	**Adriamycin**	**Epirubicin**
CMF/oral	100 p.o.	40 i.v.	600 i.v.		
(6 cycles)	d1–14	d1, d8	d1, d8		
	(28d cycle)	(28d cycle)	(28d cycle)		
CMF/i.v.	600 i.v.	40–50 i.v.	600 i.v.		
(6 cycles)	d1, d8	d1, d8	d1, d8		
	(28d cycle)	(28d cycle)	(28d cycle)		
CMF 3-weekly	600–750 i.v.	40 i.v.	600 i.v.		
(5 – 8 cycles)	d1	d1	d1		
	(21d cycle)	(21d cycle)	(21d cycle)		
AC	600 i.v.			60 i.v.	
(4 – 6 cycles)	d1			d1	
	(21d cycle)			(21d cycle)	
Adriamycin/CMF	600 i.v.	40 i.v.	600 i.v.	75 i.v.	
(4 cycles A;	d1	d1	d1	d1	
8 cycles CMF)	(21d cycle)	(21d cycle)	(21d cycle)	(21d cycle)	
FEC	600 i.v.		600 i.v.		60–75 i.v.
(6 cycles)	d1		d1		d1
	(21d cycle)		(21d cycle)		(21d cycle)
					
					
NEAT	600 i.v.	40 i.v.	600 i.v.		100 i.v.
(4 cycles epirubicin;	d1, d8	d1, d8	d1, d8		d1
4 cycles CMF)	(28d cycle)	(28d cycle)	(28d cycle)		(21d cycle)
Adriamycin (4 cycles)				75 i.v.	
				d1	
				(21d cycle)	
					
CAF	600 i.v.		600 i.v.	60 i.v.	
(6 cycles)	d1		d1	d1	
	(21d cycle)		(21d cycle)	(21d cycle)	
Epirubicin/ CMF	750 i.v. d1	50 i.v. d1	600 i.v. d1		100 i.v. d1
(6 cycles)	(21d cycle)	(21d cycle)	(21d cycle)		(21d cycle)
Epirubicin/ CMF	600 i.v. d1	40 i.v. d1	600 i.v. d1		75 i.v. d1
(8 cycles)	(21d cycle)	(21d cycle)	(21d cycle)		(21d cycle)
Epirubicin/ CMF	600 i.v. d1	40 i.v. d1	600 i.v. d1		75 i.v. d1
(12 cycles)	(28d cycle)	(28d cycle)	(28d cycle)		(28d cycle)
					

d=day. Other abbreviations same as in Table 3a.

), with 61% of patients receiving CMF-based regimens and 39% receiving anthracycline-based regimens. The median age of patients receiving CMF- and anthracycline-based regimens was 49.4 and 48.1 years, respectively.

### Incidence of neutropenic events – all patients

Of the 422 patients, 121 (29%) experienced at least one neutropenic event (Table 4

**Table 4:** Incidence of neutropenic events

	**CMF-based regimens**			**Anthracycline-based regimens**						
	**CMF/oral d1 – 8**	**CMF/i.v. d1 – 8**	**CMF (3-weekly)**	**AC**	**Adriamycin/CMF**	**FEC**	**NEAT**	**Adriamycin**	**CAF**	**Epirubicin/CMF**
Patients (*n*)^a^	119	67	71	49	45	36	13	9	7	3
Neutropenic event (% patients)	28	19	41	12	49	11	54	44	29	0
Dose delay^b^										
(% patients)	19	13	38	6	40	11	46	33	0	0
Dose reduction^b^										
(% patients)	11	5	7	4	4	0	31	0	0	0
Hospitalisation^b^										
(% patients)	6	8	1	8	11	0	8	11	29	0

d=day.

a Includes patients who received G-CSF as prophylaxis (CMF-based regimens *n*=17; anthracycline-based regimens *n*=5).

b Due to neutropenia.

). The median ANC at which chemotherapy was delayed or reduced was 0.9 × 10^9^ l^−1^ (range 0.20–1.8 × 10^9^ l^−1^). Although a greater percentage of patients who experienced a neutropenic event were receiving concomitant radiotherapy, the impact of this intervention did not significantly affect the risk of neutropenic events. Of the 121 patients with a neutropenic event, 44% received concomitant radiotherapy compared with 33% of the 301 patients who did not experience a neutropenic event (*P*=NS). Overall, out of the 422 patients in the audit, 17% of patients did not achieve 85% of their planned DI; due to neutropenia in 11% of patients. The mean RDI was significantly lower (*P*<0.01) in those who experienced a neutropenic event compared with those patients who did not.

A small percentage (5.2%) of patients received a haematopoietic growth factor, which in all cases was G-CSF. Granulocyte colony-stimulating factor was administered either to hospitalised patients as treatment for febrile neutropenia (1.7%), or to nonhospitalised patients who had experienced a neutropenic event in a previous cycle (as secondary prophylaxis) to avoid further complications (3.6%). Granulocyte colony-stimulating factor was given for between 1 and 8 days. On average, G-CSF was given for longer (6.3 *vs* 1.4 days) and over more cycles (2 *vs* 1 cycle) when administered as secondary prophylaxis. Owing to the small number of patients receiving G-CSF, further evaluation of this group was not considered appropriate.

As the majority of patients involved in the audit received CMF- or anthracycline-based regimens, further evaluation was performed specifically on these two groups of patients.

### Incidence of neutropenic events – CMF-based regimens

Overall, 29% of patients receiving CMF-based regimens experienced a neutropenic event. Table 4 shows the incidence of neutropenic events in patients receiving specific CMF-based regimens. Dose delay was the most frequent (occurring in 23% of patients) followed by dose reduction (8% of patients) and hospitalisation (5% of patients). Dose delay due to neutropenia was also the most common neutropenic event for each individual CMF-based regimen. The incidence of neutropenic events was highest in those receiving 3-weekly intravenous CMF.

**Figure 1:**
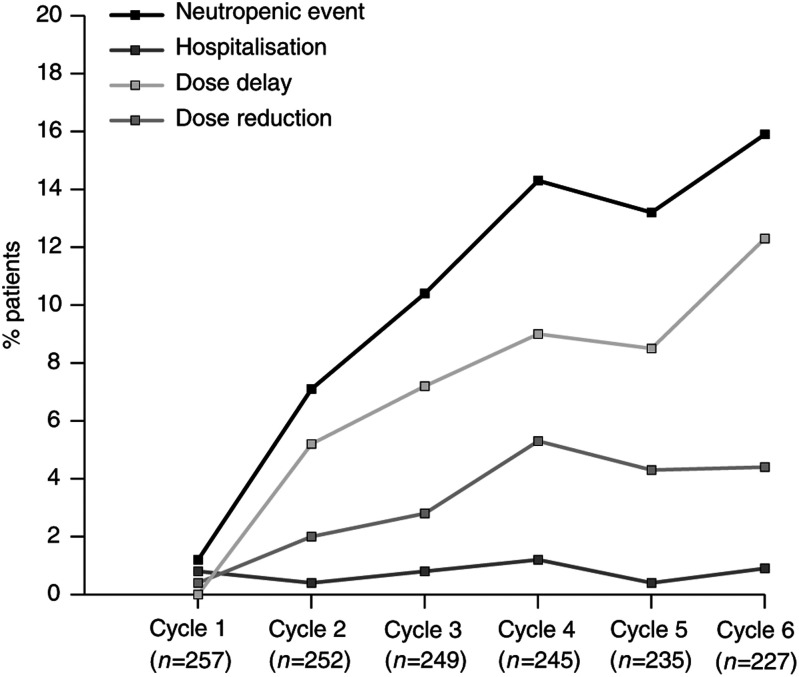
Incidence of neutropenic events by cycle: CMF-based regimens.

The incidence of neutropenic events by cycle in patients receiving CMF-based regimens is shown in Figure 1

, with the overall incidence of neutropenic events tending to increase with cycle number. For all cycles, the majority of neutropenic events were dose delays and the incidence of hospitalisation was low. The median cycle delay due to neutropenia was 7 days and the median dose reduction due to neutropenia was 26.8%.

**Figure 2:**
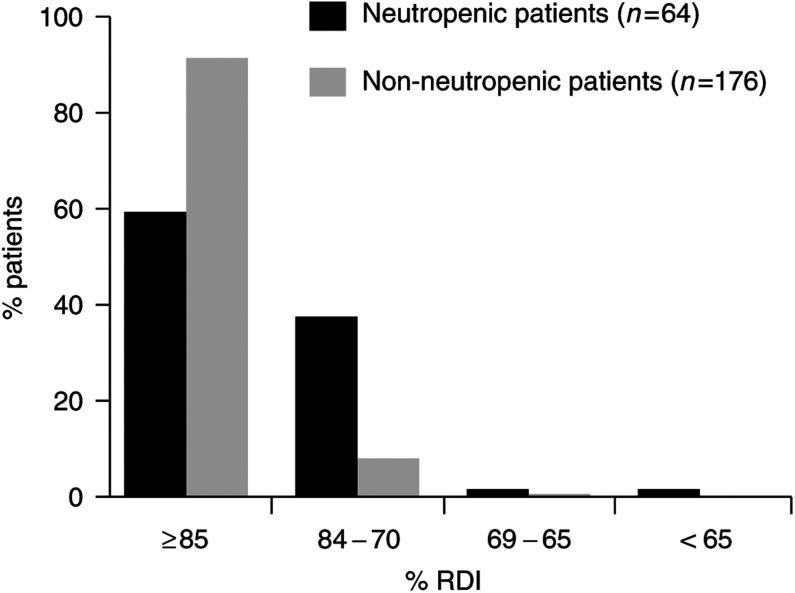
Impact of neutropenic events on RDI: CMF-based regimens.

The impact of neutropenic events on RDI for patients receiving CMF-based regimens is shown in Figure 2

. Patients receiving G-CSF as prophylaxis (*n*=17) were excluded from this analysis. Patients who experienced neutropenic events received a significantly lower mean RDI than those who did not experience neutropenic events (86.9 *vs* 95.7%, *P*<0.01). The median (range) RDI was 88% (58.5–125.9%) and 98.8% (67–110%) for patients who experienced and who did not experience neutropenic events, respectively. Almost 40% of patients with neutropenic events did not achieve 85% of the planned DI, compared with 9% of patients in the group with no complications. However, only 3% of patients experiencing neutropenic events were not able to achieve 70% of the planned RDI.

### Incidence of neutropenic events – anthracycline-based regimens

Overall, 28% of patients receiving anthracycline-based regimens experienced a neutropenic event. Dose delay was again the most common event (occurring in 21% of patients), followed by hospitalisation (8.5% of patients) and dose reduction (5.5% of patients).

Table 4 shows the incidence of neutropenic events recorded in patients receiving specific anthracycline-based regimens. For all regimens except CAF, dose delay was the most frequent neutropenic event. Where dose delays and reductions were not employed, as for CAF, the incidence of hospitalisation was high. The incidence of neutropenic events was highest in those receiving the NEAT protocol (epirubicin 100 mg m^−2^ followed by CMF).

**Figure 3:**
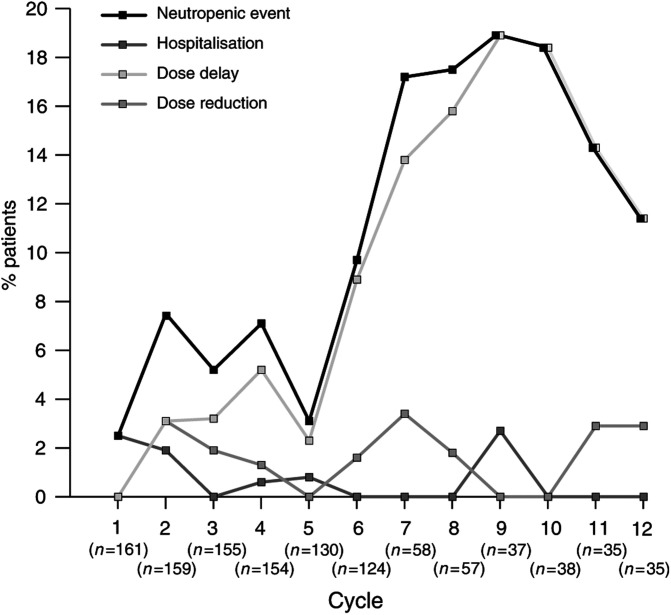
Incidence of neutropenic events by cycle: anthracycline-based regimens.

The incidence of neutropenic events by cycle is shown in Figure 3

. The incidence of neutropenic events appears to increase significantly after cycle 5. This represents patients receiving NEAT (eight cycles) and adriamycin/CMF (12 cycles), suggesting that most events occur in patients on CMF who have previously been exposed to anthracyline. As with CMF-based regimens, the median cycle delay due to neutropenia was 7 days, whereas the median dose reduction due to neutropenia was slightly lower than for CMF at 20%.

**Figure 4:**
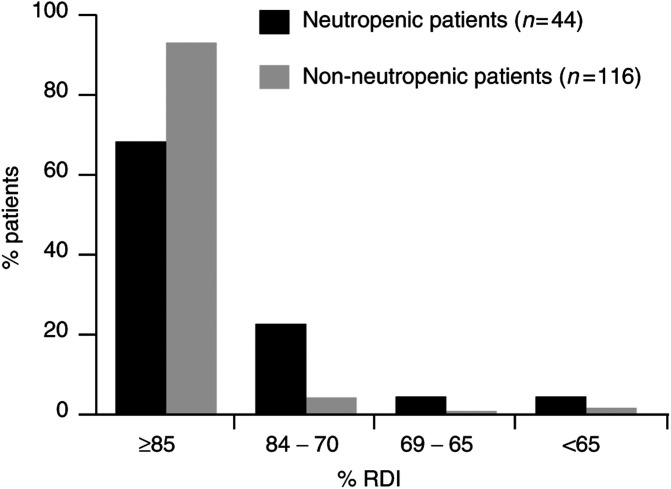
Impact of neutropenic events on RDI: anthracycline-based regimens.

The overall impact of neutropenic events on RDI for patients receiving anthracycline-based regimens is shown in Figure 4

. Patients who received G-CSF as prophylaxis (*n*=5) were excluded from this analysis. Patients who experienced neutropenic events received a significantly lower mean RDI than those who did not experience neutropenic events (87.4 *vs* 96.3%, *P*<0.01). The median (range) RDI was 91.1% (56.7–111%) and 99.3% (66.8–122%) for patients who experienced and who did not experience neutropenic events, respectively. Of those patients with neutropenic events, 32% did not achieve 85% of the planned DI, compared with 7% of patients in the group with no events. Importantly, 9% of patients experiencing neutropenic events failed to receive 70% of the planned DI (Figure 4).

### Risk of subsequent neutropenic events after a first event

For both CMF- and anthracycline-based regimens, the risk of experiencing a neutropenic event was higher in those patients who had already experienced an event in an earlier cycle (Table 5

**Table 5:** Risk of subsequent neutropenic events after a first event

**Regimen**	**% patients experiencing at least one neutropenic event**	**% patients experiencing a neutropenic event after a first event**
CMF-based (*n*=257)	29	56
Anthracycline-based (*n*=165)^a^	28	72

a In total, 162 patients receiving anthracycline-based regimens and three patients receiving either epirubicin, epirubicin/cyclophosphamide or docetaxel.

). For CMF-based regimens, the risk increased almost two-fold to a 1 : 2 risk, whereas for anthracycline-based regimens, the risk increased almost 2.6-fold to a 3 : 4 risk.

## DISCUSSION

To our knowledge, this is the first time that the incidence of neutropenic events in patients with primary breast cancer undergoing adjuvant chemotherapy has been assessed in the UK. This was not a controlled clinical trial, but an audit designed to provide an indication of what actually occurred in clinical practice. As such, we believe it provides an informative insight into our current practice in the chemotherapeutic management of early breast cancer.

A wide variety of chemotherapy regimens is currently in use across the UK for the adjuvant treatment of breast cancer, with CMF- and anthracycline-based regimens predominating. These findings are similar to those of the ChemoInsight Project, a large ongoing US retrospective analysis of 20 106 patient records in which CMF, AC and CAF have so far been the most common regimens recorded, with CMF being used in 44.2% of patients (Hayes, 2001).

The audit indicates that neutropenic events occur frequently during adjuvant chemotherapy for primary breast cancer, occurring in approximately 30% of patients. Dose delay was found to be the most common strategy used to cope with neutropenic events, followed by dose reduction. These findings are again in accordance with those of the ChemoInsight Project, which identified 43.1% of 20 106 patients experiencing dose delays, compared with 25.7% experiencing dose reductions (Hayes, 2001).

A median cycle of 7 days seen in the audit reflects our current practice of delaying chemotherapy for a week in those patients whose blood counts have failed to recover adequately from prior chemotherapy. No doubt this reflects a convenience factor relating to work load and clinic appointment times, and the high frequency of dose delays may have been the reason for a low frequency of hospitalisations. Strategies that permit shorter delays by rechecking blood counts after 2–3 days would probably ameliorate the impact of dose delay on RDI. However, this may only be possible in nurse-led clinics, or alternatively, in open-access chemotherapy units where patients attend daily until blood counts returned to normal, at which stage chemotherapy could be administered.

Dose delays and dose reductions have a significant impact on RDI (Longo *et al*, 1991), and consequently neutropenic events were found to have a significant impact on the ability to deliver planned DI in the audit to such an extent that survival may have been affected. For both CMF- and anthracycline-based regimens, a high proportion of patients with neutropenic events did not achieve 85% of the planned DI compared with the group with no events. Importantly, 3 and 9% of patients receiving CMF- and anthracycline-based regimens, respectively, who experienced a neutropenic event were not able to receive 70% of the planned dose.

For CMF-based treatment regimens, the incidence of neutropenic events (predominantly dose delays) was highest with CMF 3-weekly regimen. A higher incidence of dose delays due to haematological toxicity in the CMF 3-weekly *vs* classical CMF regimens has also been reported by Engelsman *et al* (1991). It is unclear why more events are seen with CMF 3-weekly when the chemotherapy DI is lower than classical i.v. Day 1, Day 8 CMF, but it is possible that the patients who received this regimen attended less experienced centres who were less aggressive in their dosing schedule. This regimen was also prescribed more often in district general hospitals, where there may be fewer available beds. Patients may therefore have had their chemotherapy delayed/reduced in order to avoid hospitalisation.

For patients receiving anthracycline-based regimens, the incidence of dose modifications due to neutropenic events was increased in the higher anthracycline dose protocols, for example, the NEAT protocol. Where delays and reductions have apparently been completely avoided, that is, in the CAF group, the hospitalisation rate is much higher. The incidence of neutropenic events found with FEC was surprisingly low. This may in part be due to the relatively lower doses of epirubicin administered over the first four cycles in FEC *vs*, for example, the higher doses administered over the first four cycles in NEAT. Higher doses of epirubicin have been associated with a significantly higher incidence of grade 3–4 neutropenia in women with poor prognosis, node-positive breast cancer, although the higher doses have also been associated with improved outcome (The French Adjuvant Study Group, 2001). However, as noted earlier, many of the neutropenic events occured during the CMF phase of the NEAT regimens.

Our findings also indicate that patients experiencing one neutropenic event are much more likely to experience further events in later cycles. This has been previously reported by Crawford *et al* (1991) in patients receiving CAE chemotherapy for small-cell lung cancer. Patients who have already experienced a neutropenic event, and in whom maintenance of DI is important, may benefit from haematopoietic growth factors such as G-CSF when given as secondary prophylaxis (American Society of Clinical Oncology, 1994, 1996; Ozer *et al*, 2000). In breast cancer patients experiencing insufficient neutrophil recovery during CMF chemotherapy, a 10-day course of G-CSF has been shown to enhance neutrophil recovery, and help maintain scheduled chemotherapy DI (de Graaf *et al*, 1996). However, from this audit it appears that the administration of G-CSF to limit the impact of neutropenic events occurs infrequently (<6%), much less than European and American counterparts where RDI is also higher (Hayes, 2001). Many clinicians may not perceive neutropenia to be an issue when administering adjuvant chemotherapy, and it is unlikely that, at the time, much consideration was given to the effects of these strategies on RDI and OS, when survival is such a distant end point. As always, cost issues are likely to play a major role in limiting the use of G-CSF, but there is increasing evidence that the costs of using G-CSF may be offset by reducing the cost of delays and hospitalisations (Seifeldin and Schultz 1998; Silber *et al*, 1998a,1998b).

In conclusion, neutropenic events have a significant impact on the ability to deliver planned chemotherapy DI in the adjuvant treatment of breast cancer, and these events may therefore have an impact upon patient survival. Further evaluation of the cost effectiveness of interventions such as G-CSF that limit neutropenic complications should be performed in order that such strategies, if cost effective, are not withheld on the basis of acquisition costs alone. Many clinicians may not be aware of the extent to which dose modifications impact upon overall DI, and should bear in mind their potential impact on survival when considering the best strategy to maximise delivery of chemotherapy.

## Appendix

**The UK Breast Cancer Neutropenia Audit Group consisted of the following investigators:** S Hima, Jersey General Hospital, Jersey; D Spooner, Dudley Road Hospital, Birmingham; P Barrett-Lee, Velindre Hospital, Cardiff; R Coleman, Weston Park Hospital, Sheffield; M Lind, A Maraveys, A Chaterverdi, Princes Royal Hospital, Hull; J Barrett, Royal Berkshire Hospital, Reading; P Simmonds, Southampton General Hospital, Southampton; N Davidson, North Middlesex Hospital, Middlesex; R Pettengell, J Mansi, St George's Hospital, London; I Smith, Royal Marsden Hospital, London.
